# Supplementary material for: Exploring the use of mobile health among patients with cardiometabolic and respiratory chronic diseases in primary care nursing: a cross-sectional study
Source: PeerJ. 2025 Oct 2;13:e20130. doi: 10.7717/peerj.20130 (PMC12497400; doi:10.7717/peerj.20130)
Supplement: Supplemental Information 2 [file peerj-13-20130-s002.pdf]

Table S1

Supplementary data for:

(Original Version)

**FORMULARIO: "Uso de los sistemas de salud móvil (mHealth) por pacientes con enfermedades crónicas en la consulta de enfermería de Atención primaria"**
**DATOS SOCIODEMOGRÁFICOS Y CLÍNICOS**
**3 últimas cifras de su SIP[a] ____________________ Centro de Salud: ______________________________________**
**Sexo: □ Hombre □ Mujer Año de nacimiento: ______________________**
**Nº de medicamentos diarios: __________ Nivel estudios: □ Sin estudios □ Primarios □ Secundaria o superior**
**Peso: ___________ Kg Altura: _____________cm**
**Tensión Arterial: ________ / ________ mmHg Glucemia digital: ___________ mg/dL**
**Diagnósticos médicos: □ Diabetes □ HTA[b] □ EPOC[c] □ Dislipemia □ Obesidad**
**Nº Consultas Enfermería crónicos presenciales Anuales: □ 0 □ 1 □ 2 □ 3 □ 4 □ +5**
**Nº consultas Enfermería crónicos telefónicas Anuales: □ 0 □ 1 □ 2 □ 3 □ 4 □ +5**
**Inclusión en algún programa Especializado: □ NO □ ICC[d] □ PCC[e] □ Paliativos**
**Grado cronicidad: □ G0[f] □ G1[g] □ G2[h] □ G3[i]**

CUESTIONARIO A REALIZAR AL PACIENTE (Marque con un X la/las respuesta/s)

1. ¿Cómo valora su estado de salud?

□ Muy buena □ Buena

□ Regular □ Mala

□ Muy mala

2. ¿Cuánto tiempo que lleva utilizando teléfono móvil?

□ No utilizo teléfono móvil

□ Más de 5 años □ Más de 10 años

□ Más de 15 años □ Más de 20 años

3. ¿Se considera hábil en el manejo de las aplicaciones móviles?

□ SI

□ NO

□ Regular

4. ¿Utiliza Aplicaciones móviles a diario?

(Por ejemplo, redes sociales, mensajería instantánea, internet, etc.)

□ Sí, mensajería, redes sociales, internet

□ Sí, solo mensajería

□ NO utilizo ninguna App (pasar a Pregunta nº 7)

5. ¿Utiliza o ha utilizado algún dispositivo o aplicación móvil aplicada a su salud?

□ SI

□ NO

5.1. Si su respuesta fue SÍ, ¿Cuál utiliza o ha utilizado?

____

(Ejemplos: GVA Salud, +Salud, Mostrador virtual MS, Salud iPhone, App Coronavirus, apps deportivas)

5.3. ¿Para qué la utiliza o ha utilizado?

□ Control y seguimiento COVID

□ Realizar actividad física

□ Control de HTA

□ Control diabetes

□ Control Peso corporal / Obesidad

□ Control Alimentación

□ Control medicación / Pastillero electrónico

□ Gestión citas / dudas administrativas

5.2. ¿Cuándo ha empezado a utilizarlas?

□ Antes de la Pandemia COVID-19 □ A raíz de la Pandemia COVID-19.

6. ¿Ha utilizado alguna aplicación móvil relacionada con la COVID-19?

□ SI

□ NO

6.1. Si su respuesta fue SÍ, ¿Para que la ha utilizado?

□ Gestión de la exposición o rastreo de contactos

□ Telemonitorización de signos vitales

□ Búsqueda de información

□ Gestiones administrativas. Gestión incapacidades

7. ¿Utilizaría una App móvil para realizar un seguimiento de sus enfermedades crónicas con el equipo de AP?

□ SI □ NO

7.1. Si su respuesta fue NO, ¿Por qué?:

□ Prefiero Consulta Presencial

□ Prefiero Consulta telefónica

□ Tendría dificultades en el manejo de app de salud.

7.2. Si su respuesta fue SÍ, ¿Cuál cree que sería su uso?

□ Evitaría visitas innecesarias a Urgencias

□ El equipo de atención primaria podría realizar un seguimiento de mis cifras de TA, Glucemia, Saturación O2…

□ Podría realizar seguimiento de mis medicamentos

□ Gestión de Agendas y citas

□ Podría contactar más rápido con el Centro de salud

8. ¿Suele buscar información sanitaria sobre tratamientos, patologías o cuidados en salud en internet?	8.1. Si su respuesta fue SÍ, ¿Dónde suele buscar?
□ SI<br>□ NO	□ Google o buscadores de internet<br>□ Páginas de organismos oficiales<br>□ Redes sociales<br>□ Apps especializadas en Salud

[a]**SIP**: Sistema de información poblacional

[b]**HTA**: Hipertensión Arterial

[c]**EPOC**: Enfermedad Pulmonar Obstructiva Crónica

[d]**ICC**: Insuficiencia Cardíaca Congestiva

[e]**PCC**: Pacientes Crónico Complejo

[f]**G0**: Grado 0, personas sanas o con problemas agudos

[g]**G1**: Grado 1, paciente con factores de riesgo

[h]**G2**: Grado 2, paciente con complejidad crónica moderada

[i]**G3**: Grado 3, paciente con alta complejidad crónica o en cuidados paliativos

(English translation)

FORM: "Use of mobile health (mHealth) systems by patients with chronic diseases in primary care nursing consultations"	
**SOCIODEMOGRAPHIC AND CLINICAL DATA**	
**Last 3 digits of your HIN[a] number** ___________________	**Health Center:** ____________________________________
**Sex:** □ Male □ Female	**Year of birth:** _____________________
**Number of daily medications:** _____________	
**Education level**: □ No formal education □ Primary education □ Secondary or higher education	
**Weight:** ___________ Kg	**Height:** _____________cm
**Blood pressure:** ____________ / _____________ mmHg	**Digital blood glucose level :** ___________ mg/dL
**Medical diagnosis:** □ Diabetes □ HTN[b] □ COPD[c] □ Dyslipidemia □ Obesity	
**Number of annual in-person nursing consultations for chronic patients :** □ 0 □ 1 □ 2 □ 3 □ 4 □ +5	
**Number of annual nursing consultations for chronic patients conducted by phone :** □ 0 □ 1 □ 2 □ 3 □ 4 □ +5	
**Patient included in any specialized program**: □ No □ CHF[d] □ CCP[e] □ Palliative Care	
**Chronicity level:** □ G0[f] □ G1[g] □ G2[h] □ G3[i]	

QUESTIONNAIRE FOR THE PATIENT (Mark with an "X" the appropriate response(s)).

1. How do you rate your health status?

- □ Very good
- □ Good
- □ Regular
- □ Poor
- □ Very poor

2. How long have you been using a mobile phone?

- □ I do not use a mobile phone
- □ More than 5 years
- □ More than 10 years
- □ More than 15 years
- □ More than 20 years

3. Do you consider yourself skilled in using mobile applications?

- □ Yes
- □ No
- □ Regular

4. Do you use mobile applications daily? ***(For example, social media, instant messaging,*** *internet, etc.)*

- □ Yes, messaging, social media, internet
- □ Yes, only messaging
- □ No, I do not use any apps (skip to question No. 7)

5. Have you used or do you currently use any device or mobile application related to your health?

- □ Yes
- □ No

5.1. If your answer was YES, which one do you use or have you used?

____

(Examples: GVA Salud, +Salud, Mostrador virtual MS, Salud iPhone, COVID-19 App, Sports Apps)

5.2. *When did you start using mobile apps?*

□ Before the COVID-19 pandemic □ Due to the COVID-19 Pandemic

5.3. For what purpose do you use or have you used mobile apps?

- □ COVID-19 monitoring and follow-up
- □ Physical activity tracking
- □ Blood pressure monitoring
- □ Diabetes control
- □ Weight control / Obesity management
- □ Nutrition tracking
- □ Medication management / Electronic pillbox
- □ Appointment scheduling / Administrative inquiries

6. Have you used any mobile application related to COVID-19?

- □ Yes
- □ No

6.1. If your answer was YES, for what purpose did you use it?

- □ Exposure management or contact tracing
- □ Telemonitoring of vital signs
- □ Information search
- □ Administrative procedures (e.g. managing temporary medical leave)

7. Would you use a mobile app to monitor your chronic diseases with the primary care team?

□ Yes □ No

7.1. If your answer was NO, why?

- □ I prefer in-person consultations
- □ I prefer phone consultations
- □ I would have difficulties using a health app

7.2. If your answer was YES, how do you think it would help?

- □ To would reduce unnecessary emergency visits
- □ The Primary Care team could monitor my HTN, glucose, O_2 saturation, etc.
- □ I could track my medications
- □ Appointment scheduling and management

	□ I could contact my Health Center more quickly
**8. Do you usually search for health information about treatments, medical conditions, or care on the internet?**<br>□ Yes<br>□ No	8.1. <u>If your answer was YES, where do you usually search?</u><br>□ Google o internet search engines<br>□ Official health organization websites<br>□ Social media<br>□ Specialized health apps

[a]HIN: Healthcare Identification Number

[b]HTN: Hypertension

[c]COPD: Chronic Obstructive Pulmonary Disease

[d]CHF: Congestive heart failure

[e]CCP: Complex Chronic Patients

[f]G0: Degree 0, healthy individuals or acute problems

[g]G1: Degree 1, patient with risk factors

[h]G2: Degree 2, patient with moderate chronic complexity

[i]G3: Degree 3, patient with high chronic complexity or Palliative care
